# Supplementary material for: Structural and thermodynamical insights into the binding and inhibition of FIH-1 by the N-terminal disordered region of Mint3
Source: J Biol Chem. 2021 Oct 14;297(5):101304. doi: 10.1016/j.jbc.2021.101304 (PMC8571082; doi:10.1016/j.jbc.2021.101304)
Supplement: Supplemental Figures S1–S10 and Tables S1, S2 [file mmc1.pdf]

Supplementary Information

Structural and thermodynamical insights into the binding and inhibition of FIH-1 by the N-terminal disordered region of Mint3

Tensho Ten [1], *Satoru Nagatoishi* [2]*, *Ryo Maeda* [3], *Masaru Hoshino* [3], *Yoshiaki Nakayama* [4], *Motoharu Seiki* [5], *Takeharu Sakamoto* [6,7], *Kouhei Tsumoto* [1,2,4]*

[1] Graduate School of Engineering, The University of Tokyo, 7-3-1 Hongo, Bunkyo-ku, Tokyo 113-8656, Japan.

[2] Project Division of Advanced Biopharmaceutical Science, The Institute of Medical Science, The University of Tokyo, 4-6-1 Shirokanedai, Minato-ku, Tokyo, 108-8639, Japan.

[3] Graduate School of Pharmaceutical Sciences, Kyoto University, 46-29 Yoshida-Shimoadachi, Sakyo-ku, Kyoto 606-8501, Japan.

[4] Graduate School of Frontier Sciences, The University of Tokyo, 4-6-1 Shirokanedai, Minato-ku, Tokyo, 108-8639, Japan

[5] Division of Cancer Cell Research, The Institute of Medical Science, The University of Tokyo, 4-6-1 Shirokanedai, Minato-ku, Tokyo, 108-8639, Japan.

[6] Department of System Biology, Institute of Medical, Pharmaceutical and Health Sciences, Kanazawa University, 13-1 Takaramachi, Kanazawa, Ishikawa, 920-8640, Japan.

[7] Department of Cancer Biology, Institute of Biomedical Science, Kansai Medical University, 2-5-1 Hirakata, Osaka, 537-1010, Japan.

Table of Contents

Table S1. HDX data summary………………………………………….………..……..S3

Table S2. Thermodynamic parameters of the interaction between FIH-1 and Mint3NT/the deletion mutants…………………..…………………………………….………..……..S6

Figure S1. DSC profile of Mint3NT.………………………………………..……..S7

Figure S2. Mint3NT or FIH-1 peptides observed during sequence identification runs mapped onto the amino acid sequence of the protein …………………………..……..S8

Figure S3. ^{1}H-^{15}N HSQC spectra of ^{15}N-Mint3NT in the absence and presence of unlabeled FIH-dimer.…………………………………………………………………...S12

Figure S4. SEC-MALS profile of FIH-1.…………………………..…..……S13

Figure S5. CD spectra of Mint3NT alone (red), FIH-1 alone (blue), complex between Mint3NT and FIH-1 (black), sum of spectra of Mint3NT and FIH-1 (dark gray), spectrum of the complex minus the spectrum of FIH-1 (white gray)………………………..….S14

Figure S6. ITC profile of the interaction between FIH-1 and Mint3NTmut…..…..…….S15

Figure S7. Mutation site of L340R shown in red (PDB ID: 1MZF)..…………………...S16

Figure S8. Melting curve of FIH-1 WT and FIH-1 L340R upon analysis using DSF…..S17

Figure S9. CD spectra of FIH-1 mutants.……………..…………………….……..S18

Figure S10. Size of the FIH-1 dimer……..……………..………………….……..S19

Table S1a. HDX data summary of Figure 1c

Data Set	Mint3NT
HDX reaction details	Temperature 10 ^{o}C
HDX time course (sec)	60, 300, 600, 1800, 3600, 7200, 14400
# of peptides	150
Sequence coverage	93.78%
Average peptide length / Redundancy	16.32 / 10.88
Replicates	1

Table S1b. HDX data summary of Figure 3c

Data Set	FIH-1 WT	FIH-1 L340R
HDX reaction details	Temperature 10 ^{o}C	Temperature 10 ^{o}C
HDX time course (sec)	60, 300, 600, 1800, 3600, 7200, 14400	60, 300, 600, 1800, 3600, 7200, 14400
# of peptides	381	367
Sequence coverage	99.46%	99.46%
Average peptide length / Redundancy	13.14 / 13.54	13.04 / 12.94
Replicates	1	1

Table S1c. HDX data summary of Figure 4c

Data Set	FIH-1 (-aKG)	FIH-1 (+aKG)
HDX reaction details	Temperature 10 ^{o}C	Temperature 10 ^{o}C
HDX time course (sec)	60, 300, 600, 1800, 7200, 14400	60, 300, 600, 1800, 7200, 14400
# of peptides	259	260
Sequence coverage	93.70%	93.70%
Average peptide length / Redundancy	16.10 / 11.95	16.14 / 12.02
Replicates	1	1

Table S1d. HDX data summary of Figure 5a

Data Set	FIH-1 (-Mint3NT)	FIH-1 (+Mint3NT)
HDX reaction details	Temperature -5 ^{o}C	Temperature -5 ^{o}C
HDX time course (sec)	30, 60, 90, 120, 300, 600	30, 60, 90, 120, 300, 600
# of peptides	331	326
Sequence coverage	99.19%	99.19%
Average peptide length / Redundancy	15.11 / 13.52	15.19 / 13.38
Replicates	1	1

Table S1e. HDX data summary of Figure 6a

Data Set	Mint3NT (-FIH1)	Mint3NT (+FIH1)
HDX reaction details	Temperature 10 °C	Temperature 10 °C
HDX time course (sec)	30, 60, 90, 120, 300, 600, 1200	30, 60, 90, 120, 300, 600, 1200
# of peptides	191	155
Sequence coverage	100.00%	100.00%
Average peptide length / Redundancy	16.20 / 13.75	15.79 / 10.88
Replicates	3	3
Repeatability (avg. stddev of #D)	0.2756	0.3774

Table S2. Thermodynamic parameters of the interaction between FIH-1 and Mint3NT/the deletion mutants

	K_D (μM)	ΔG (kcal mol^{-1})	ΔH (kcal mol^{-1})	$-T\Delta S$ (kcal mol^{-1})
1-214 (NT)	0.52 ± 0.02	-8.6 ± 0.1	-32.2 ± 0.7	23.6 ± 0.8
1-117	0.65 ± 0.07	-8.4 ± 0.1	-17.9 ± 0.6	9.5 ± 0.7
1-92	9.0 ± 0.90	-6.9 ± 0.1	-14.3 ± 1.4	7.4 ± 1.4
93-117	3.0 ± 0.60	-7.5 ± 0.1	-8.7 ± 0.2	1.2 ± 0.3
93-117mut	n.d.	n.d.	n.d.	n.d.

n.d.: not determined

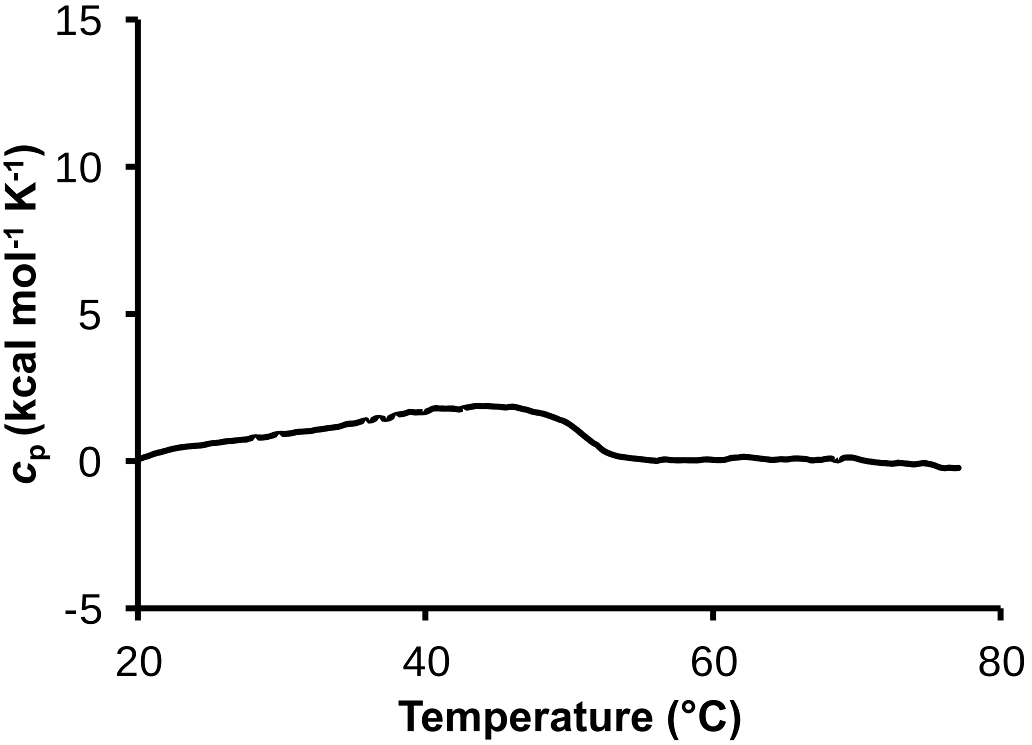

Figure S1. DSC profile of Mint3NT.

(a)

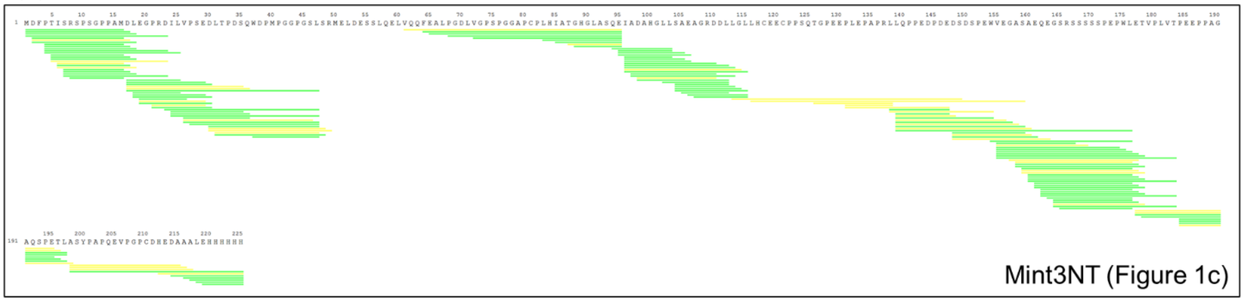

Figure S2a. Mint3NT peptides observed during sequence identification runs mapped onto the amino acid sequence of the protein in Figure 1c. Peptides are represented as green or yellow bars and aligned to their relative position along the primary sequence.

(b)

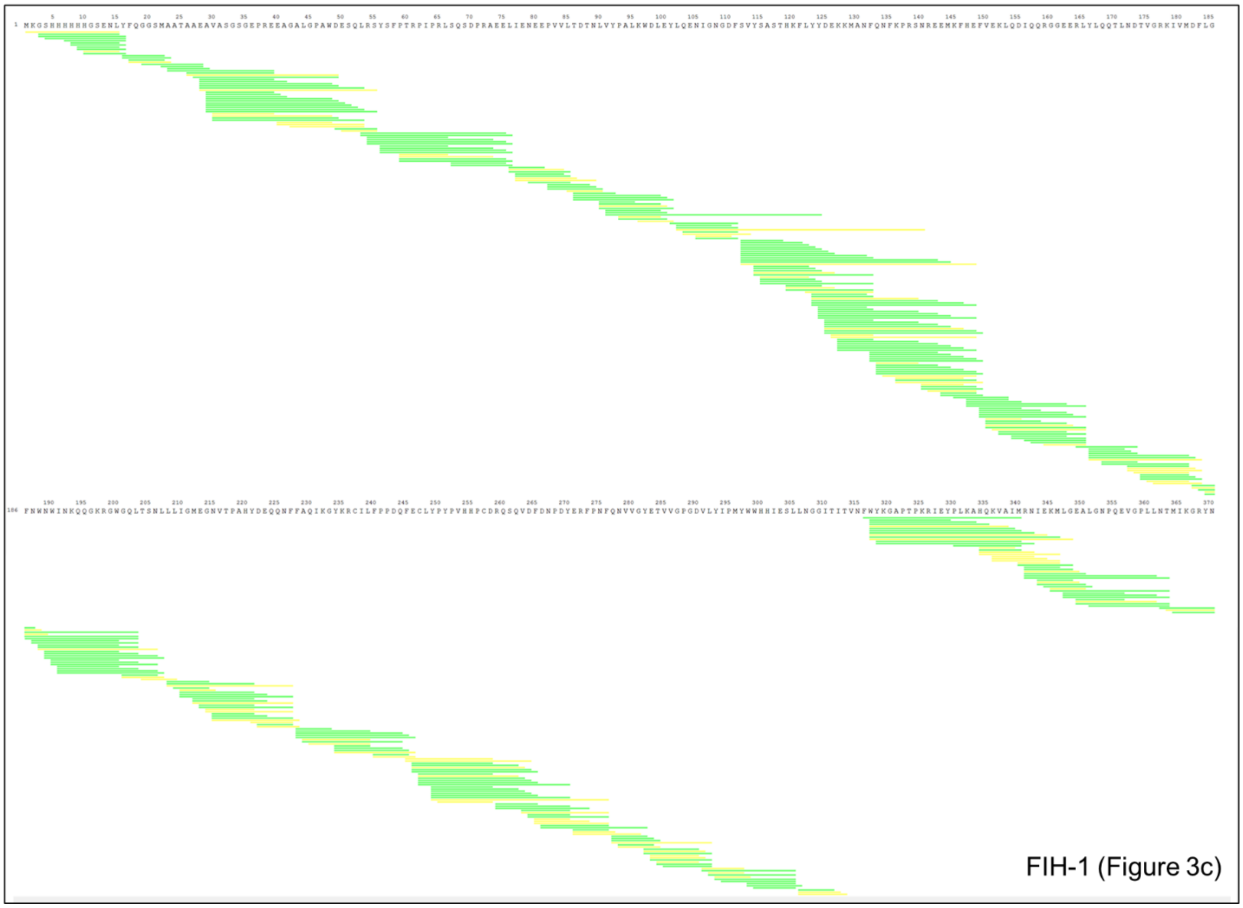

Figure S2b. FIH-1 peptides observed during sequence identification runs mapped onto the amino acid sequence of the protein in Figure 3c. Peptides are represented as green or yellow bars and aligned to their relative position along the primary sequence.

(c)

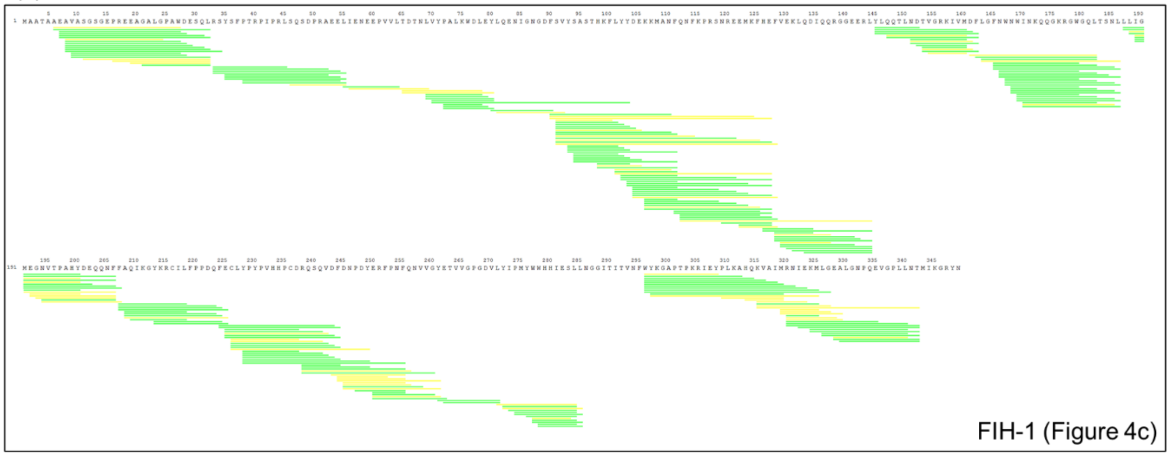

Figure S2c. FIH-1 peptides observed during sequence identification runs mapped onto the amino acid sequence of the protein in Figure 4c. Peptides are represented as green or yellow bars and aligned to their relative position along the primary sequence.

(d)

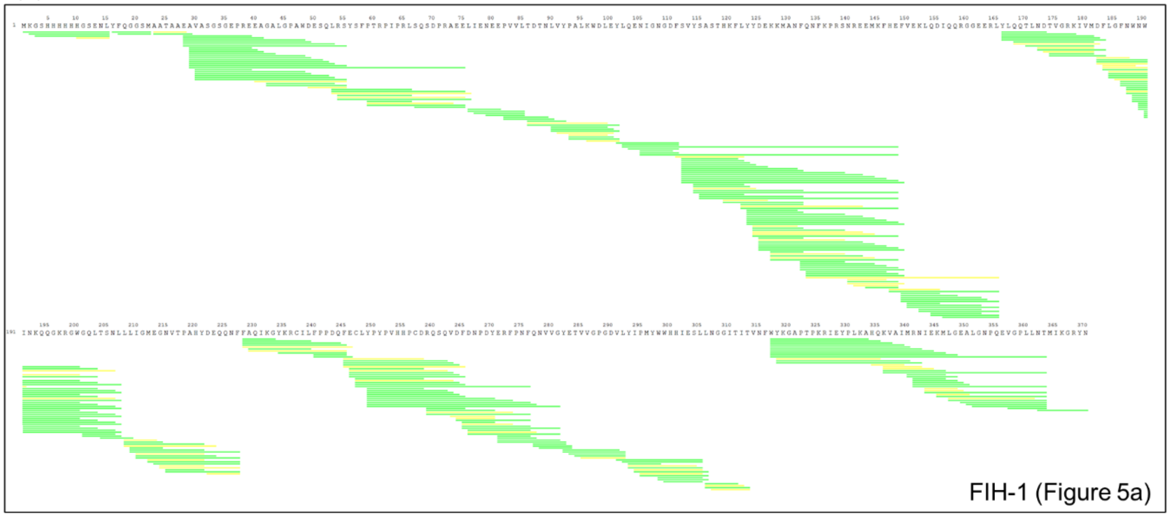

Figure S2d. FIH-1 peptides observed during sequence identification runs mapped onto the amino acid sequence of the protein in Figure 5a. Peptides are represented as green or yellow bars and aligned to their relative position along the primary sequence.

(e)

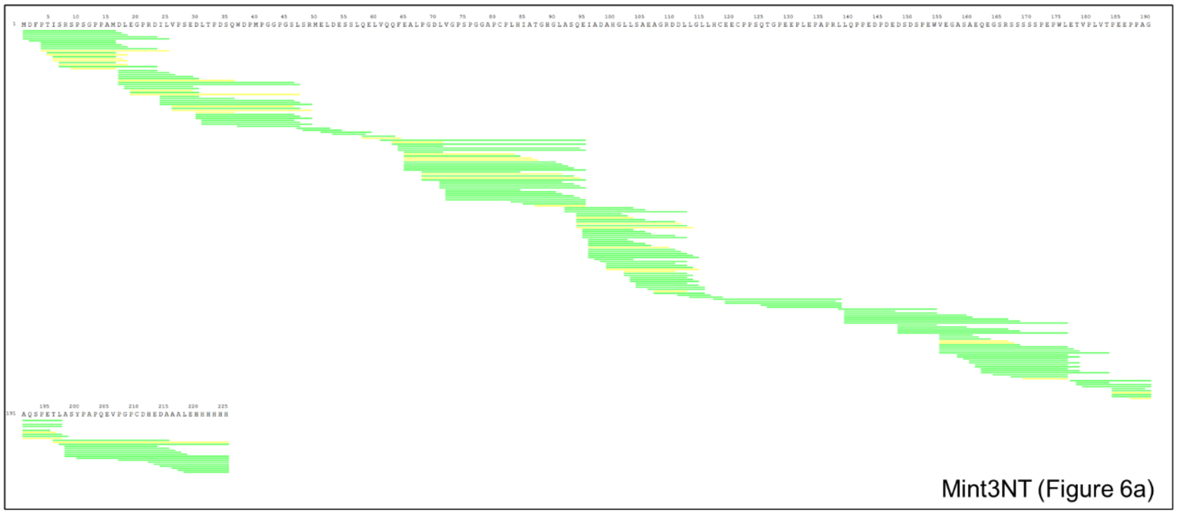

Figure S2e. Mint3NT peptides observed during sequence identification runs mapped onto the amino acid sequence of the protein in Figure 6a. Peptides are represented as green or yellow bars and aligned to their relative position along the primary sequence.

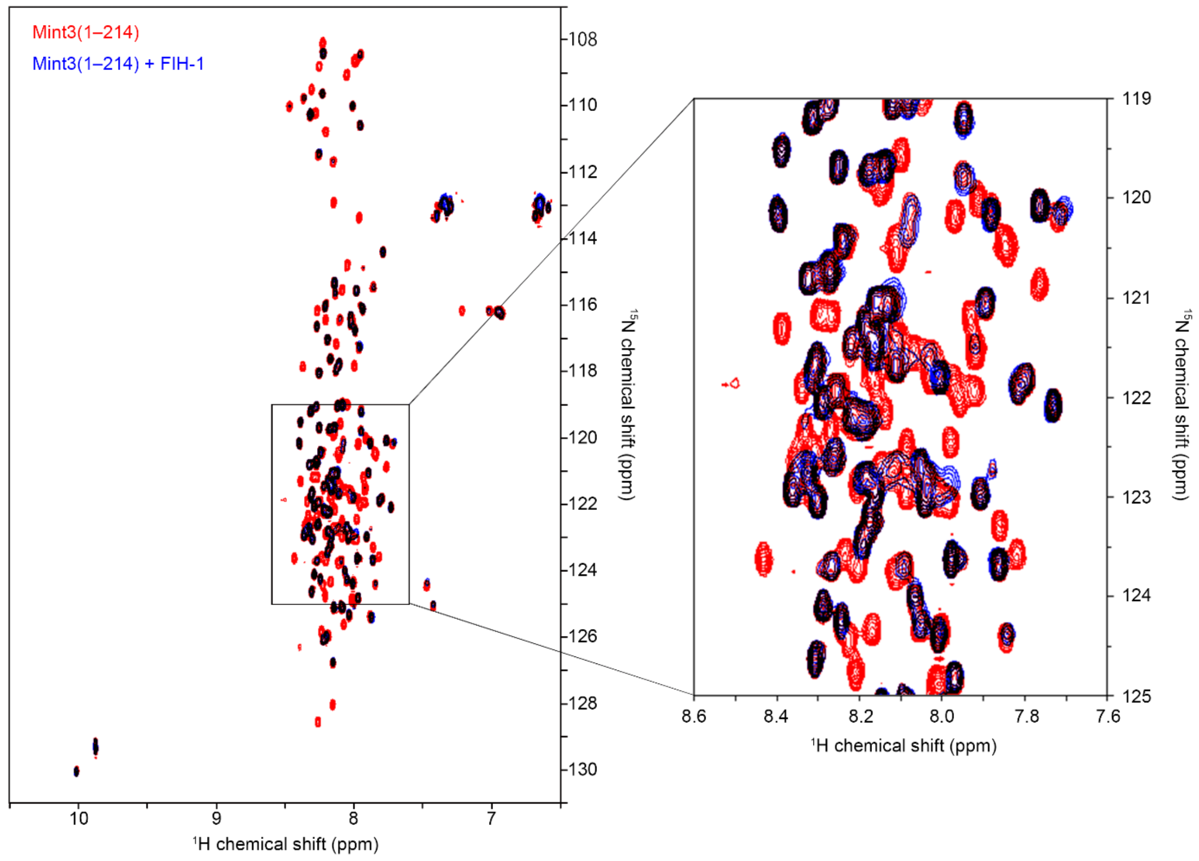

Figure S3. ^{1}H-^{15}N HSQC spectra of ^{15}N-Mint3NT in the absence (red) and presence (blue) of equimolar concentration of unlabeled FIH-1 dimer at pH 7.3 and 4°C. Note that two separately measured spectra were overlaid in "multiply blend mode", resulting in that two peaks in the same intensity and position were apparently shown in black.

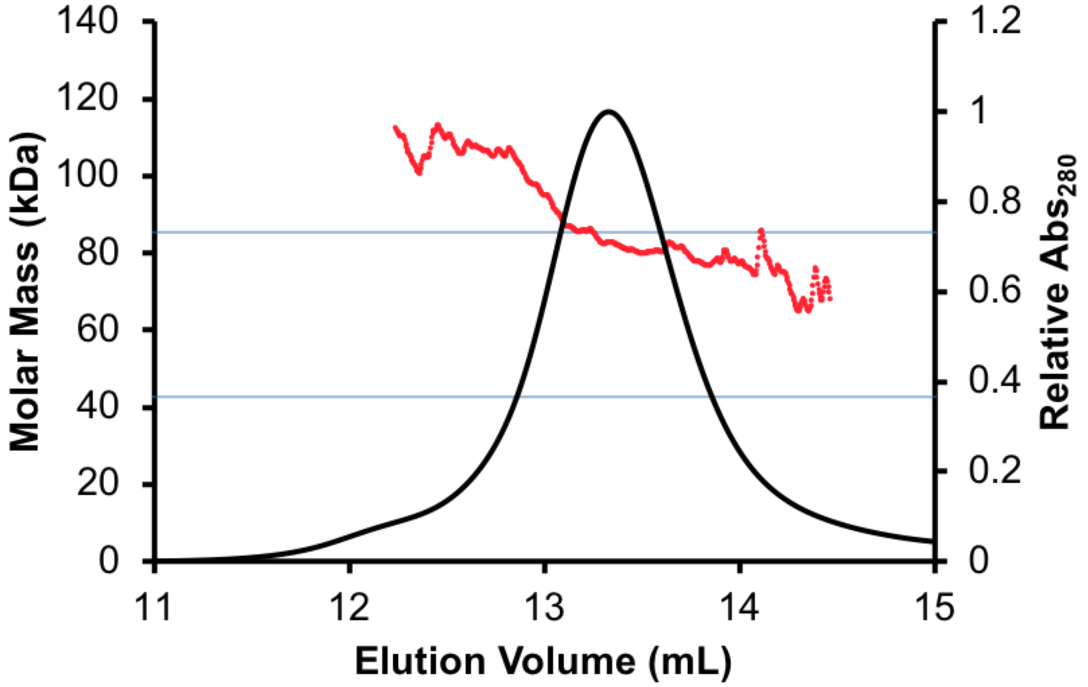

Figure S4. SEC-MALS profile of FIH-1.

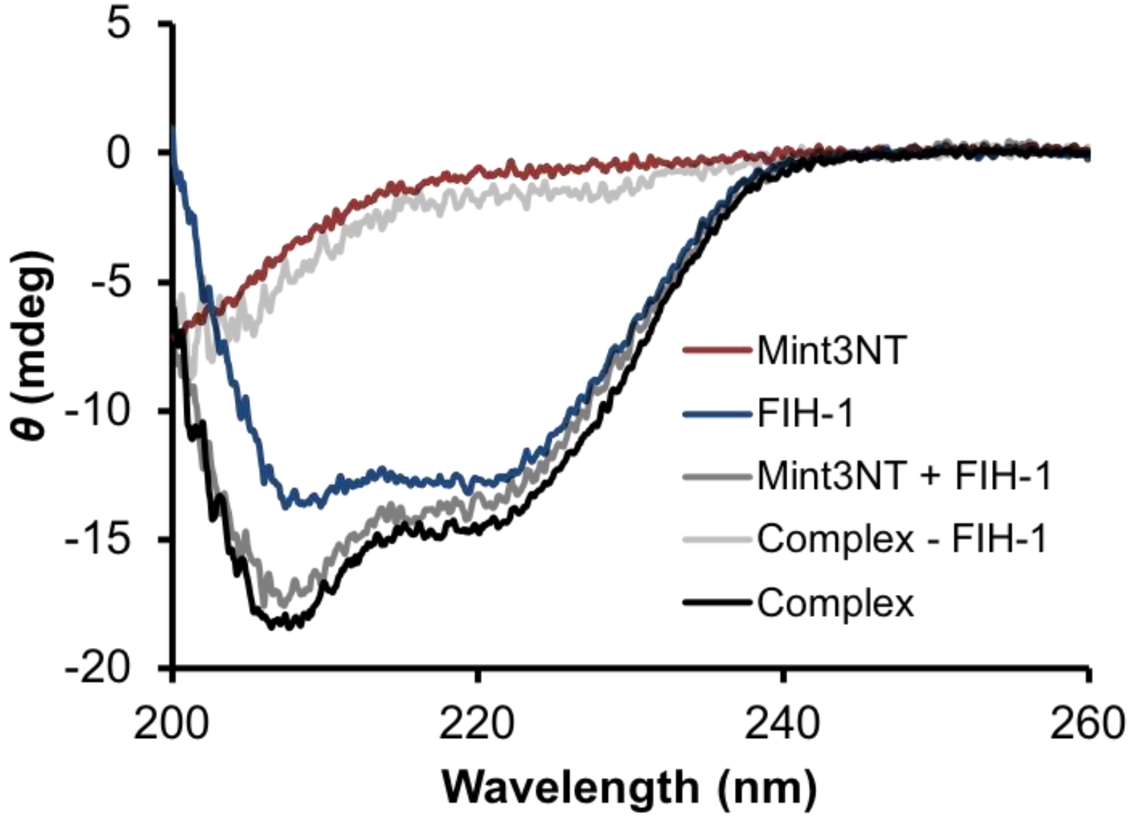

Figure S5. CD spectra of Mint3NT alone (red), FIH-1 alone (blue), complex between Mint3NT and FIH-1 (black), sum of spectra of Mint3NT and FIH-1 (dark gray), spectrum of the complex minus the spectrum of FIH-1 (white gray).

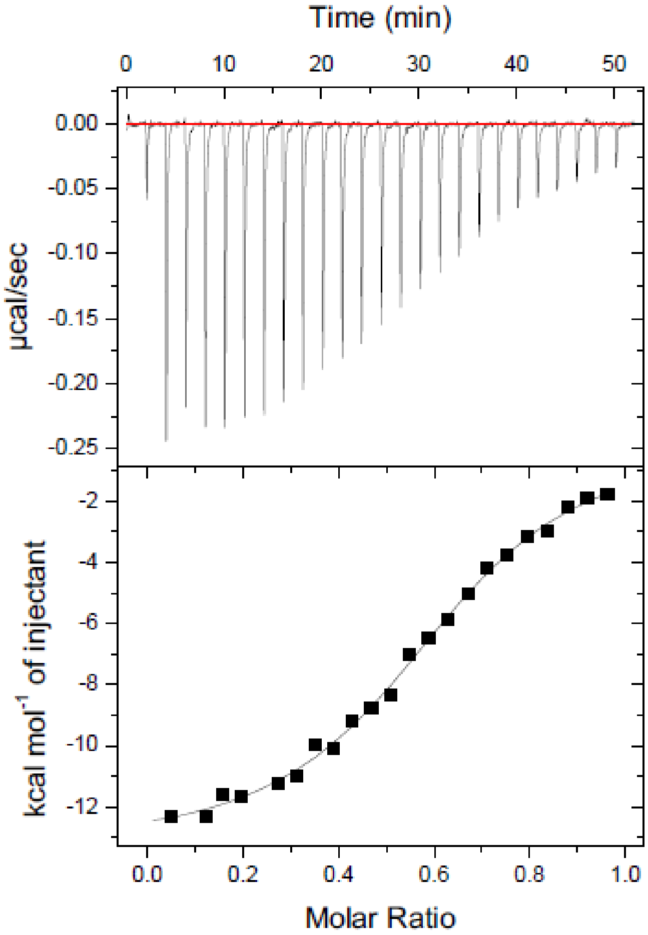

Figure S6. ITC profile of the interaction between FIH-1 and Mint3NTmut.

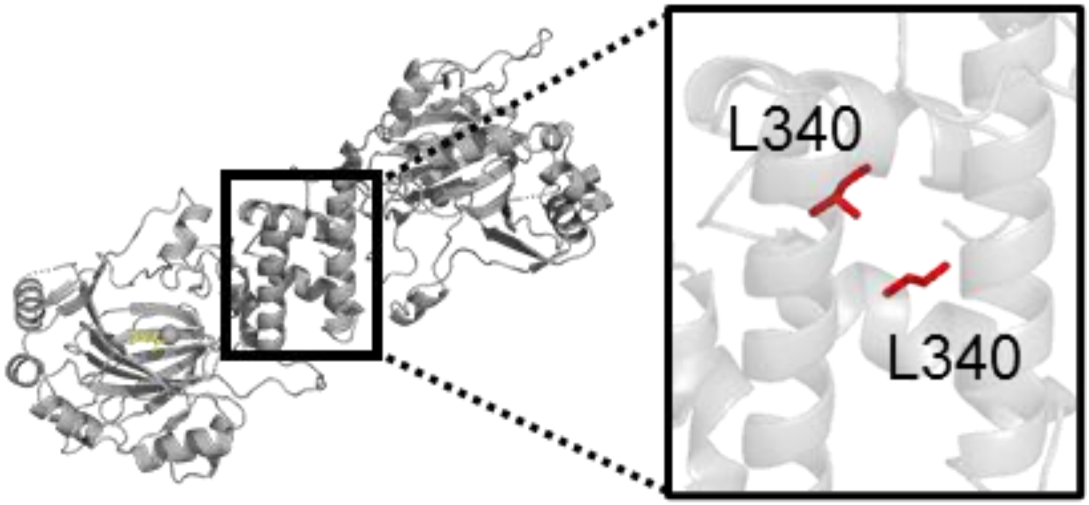

Figure S7. Mutation site of L340R shown in red (PDB ID: 1MZF).

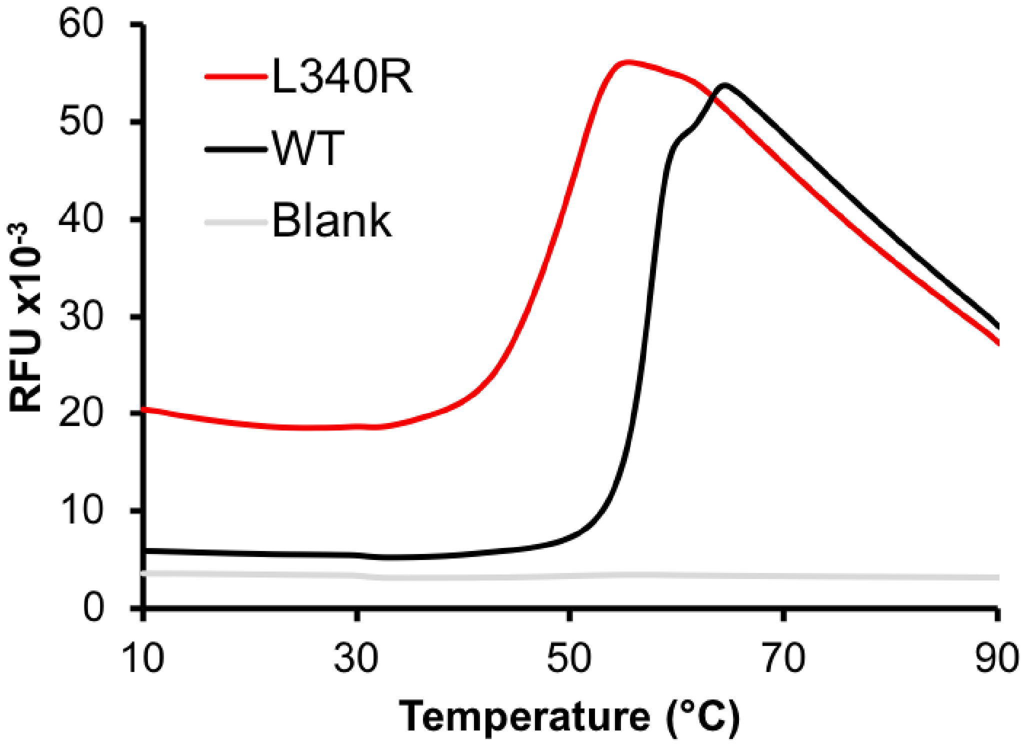

Figure S8. Melting curve of FIH-1 WT and FIH-1 L340R upon analysis using DSF.

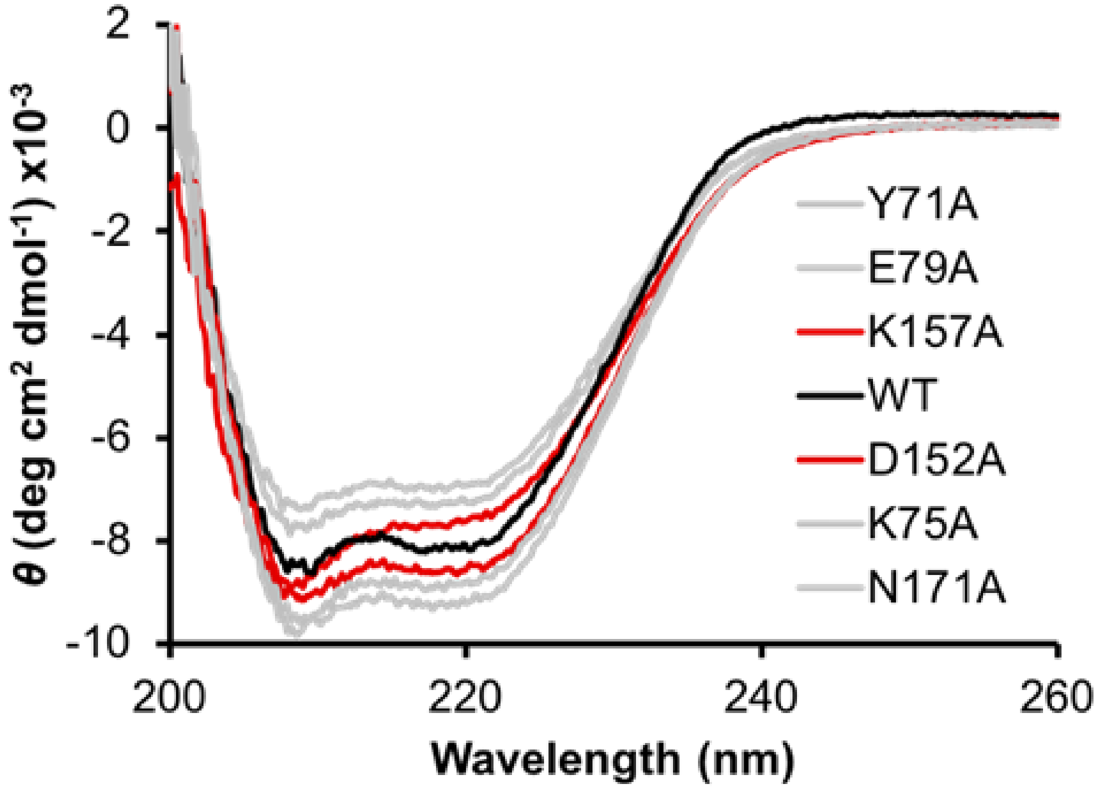

Figure S9. CD spectra of FIH-1 mutants.

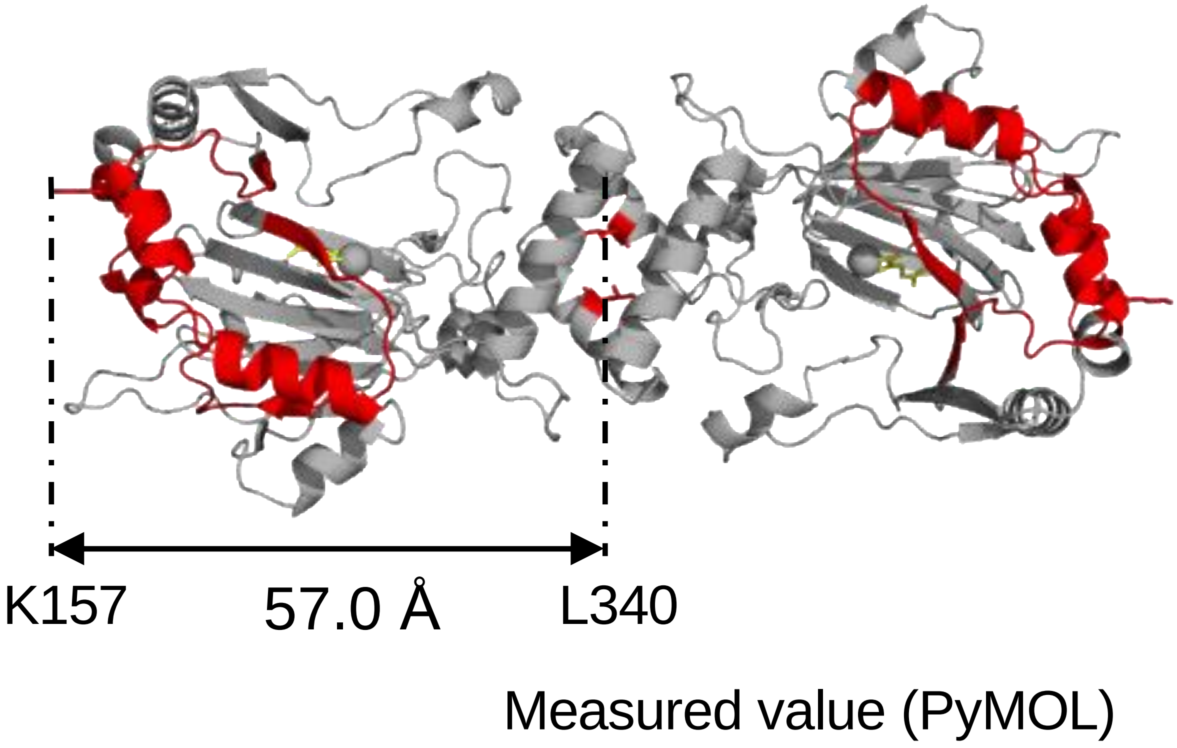

Figure S10. Size of the FIH-1 dimer calculated using Pymol software (PDB ID: 1MZF).
